# Supplementary material for: Seroprevalence of Neutralizing Antibodies in Healthy Adults, in Mexico, Against Human and Simian Adenovirus Types
Source: Viruses. 2025 Aug 29;17(9):1184. doi: 10.3390/v17091184 (PMC12474060; doi:10.3390/v17091184)
Supplement: Supplementary file 1 [file viruses-17-01184-s001.zip › viruses-3788416-supplementary.pdf]

Supplementary Figures

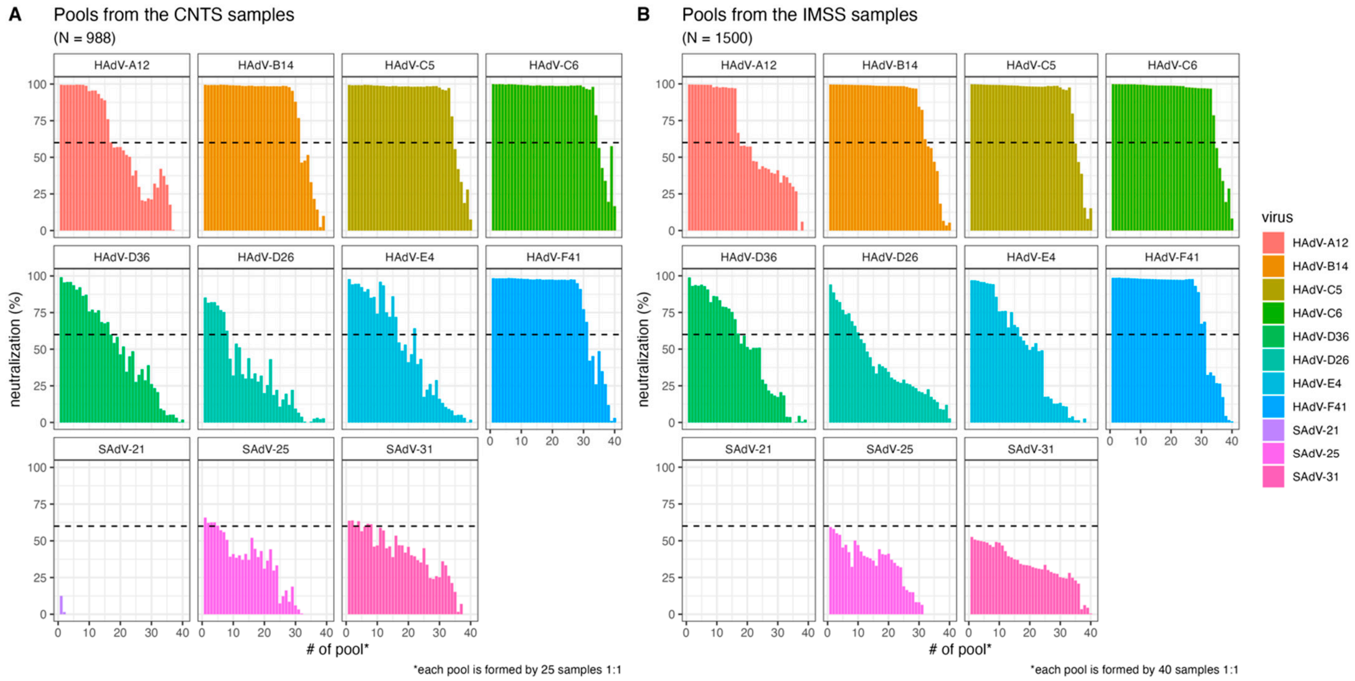

Supplementary Figure S1. Neutralization assessment of pooled samples against AdVs. Neutralization assays were performed as described in materials and methods. A) For samples from the CNTS (N=988), pools of 25 samples in equal proportions were prepared and for B) samples from IMSS (N=1500), pools of 40 samples in equal proportions were prepared, in both cases according to the ELISA absorbance values for each of the 11 analyzed AdVs. Pools above a 60% neutralization threshold were considered positive for neutralization (dashed line).

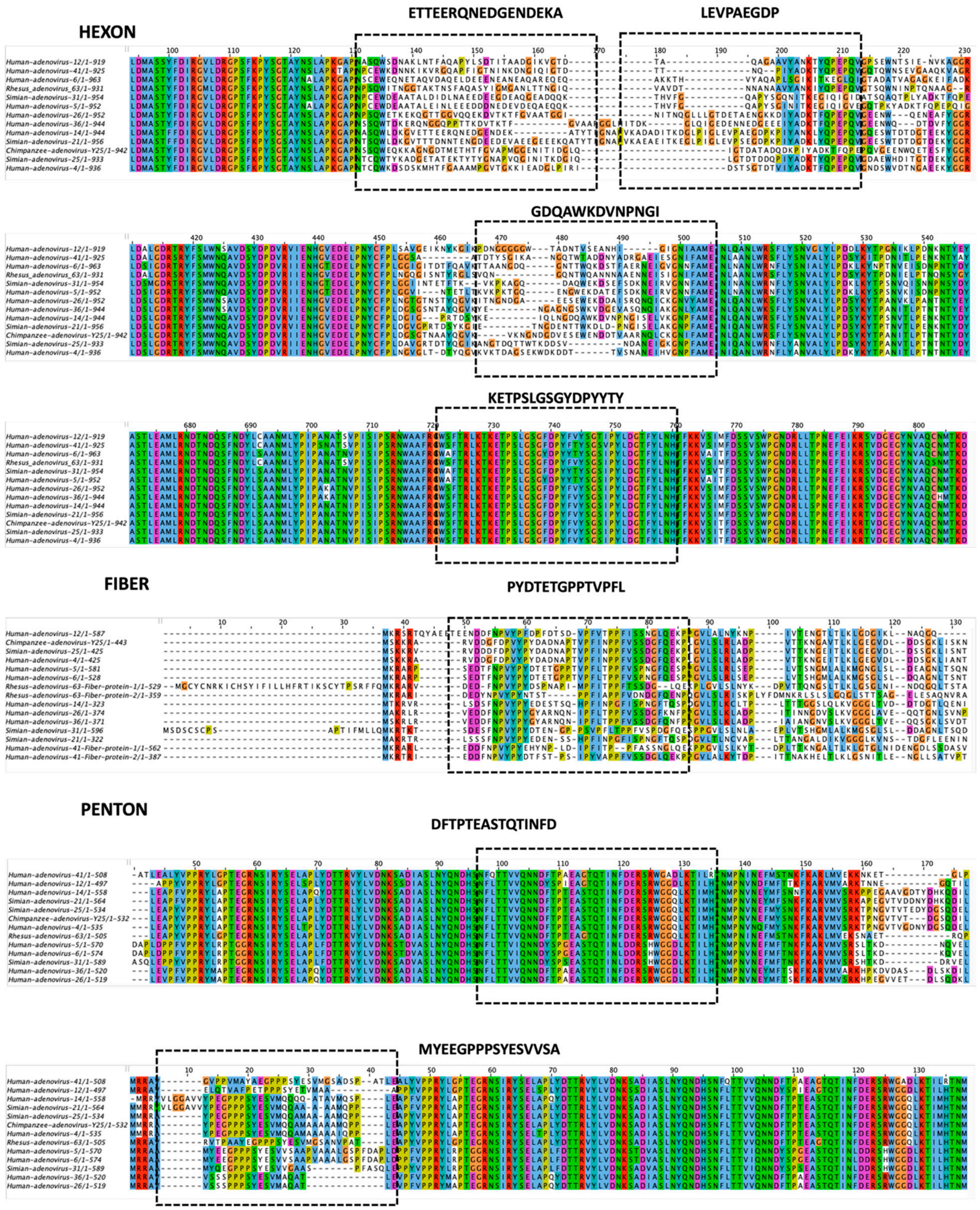

Supplementary Figure S2. Multiple sequence alignments of adenovirus structural proteins (hexon, penton, and fiber). Previously reported B-cell epitopes are highlighted in bold, and their sequence context is shown within dashed boxes. For hexon, most epitopes are located within hypervariable regions, which

nevertheless show a considerable degree of conservation across several serotypes, suggesting a basis for antibody cross-reactivity. In penton, the epitopes also map to variable regions, although their conservation is more restricted and appears to cluster by adenovirus species. For fiber, epitopes are less clearly defined, likely reflecting the requirement for protein trimerization and recognition of conformational epitopes at the knob domain.

	Seroprevalence (%)											
	N	HAdV-A12	HAdV-B14	HAdV-C5	HAdV-C6	HAdV-D36	HAdV-D26	HAdV-E4	HAdV-F41	SAdV-21	SAdV-25	SAdV-31
**NORTHEAST**												
COAHUILA	51	68.6%	94.1%	100.0%	98.0%	80.4%	5.9%	98.0%	76.5%	2.0%	98.0%	100.0%
NUEVO LEON	50	82.0%	90.0%	98.0%	98.0%	90.0%	14.0%	98.0%	90.0%	2.0%	88.0%	100.0%
TAMAULIPAS	49	77.6%	93.9%	100.0%	100.0%	69.4%	6.1%	95.9%	93.9%	0.0%	98.0%	98.0%
**NORTHWEST**												
BAJA CALIFORNIA	45	68.9%	95.6%	95.6%	88.9%	75.6%	33.3%	100.0%	95.6%	2.2%	100.0%	100.0%
BAJA CALIFORNIA SUR	50	58.0%	96.0%	100.0%	88.0%	68.0%	36.0%	98.0%	94.0%	2.0%	94.0%	100.0%
CHIHUAHUA	41	41.5%	90.2%	97.6%	97.6%	73.2%	12.2%	92.7%	87.8%	0.0%	87.8%	97.6%
DURANGO	46	67.4%	97.8%	97.8%	97.8%	89.1%	19.6%	97.8%	89.1%	13.0%	97.8%	97.8%
SINALOA	54	48.1%	96.3%	100.0%	100.0%	88.9%	35.2%	100.0%	100.0%	20.4%	98.1%	100.0%
SONORA	46	87.0%	89.1%	100.0%	100.0%	93.5%	4.3%	100.0%	91.3%	15.2%	95.7%	100.0%
**WEST**												
COLIMA	34	88.2%	94.1%	100.0%	100.0%	94.1%	2.9%	97.1%	94.1%	0.0%	97.1%	100.0%
JALISCO	51	88.2%	98.0%	100.0%	100.0%	72.5%	37.3%	100.0%	100.0%	7.8%	100.0%	100.0%
MICHOACAN	37	97.3%	100.0%	100.0%	100.0%	94.6%	40.5%	97.3%	100.0%	35.1%	100.0%	100.0%
NAYARIT	45	75.6%	97.8%	100.0%	100.0%	88.9%	2.2%	97.8%	95.6%	2.2%	93.3%	97.8%
**CENTER NORTH**												
AGUASCALIENTES	52	78.8%	100.0%	100.0%	100.0%	82.7%	46.2%	100.0%	98.1%	1.9%	100.0%	100.0%
GUANAJUATO	57	82.5%	98.2%	98.2%	98.2%	89.5%	50.9%	96.5%	84.2%	1.8%	98.2%	100.0%
QUERETARO	46	87.0%	93.5%	95.7%	95.7%	93.5%	10.9%	95.7%	89.1%	6.5%	95.7%	97.8%
SAN LUIS POTOSI	51	74.5%	90.2%	100.0%	100.0%	92.2%	13.7%	100.0%	94.1%	5.9%	94.1%	100.0%
ZACATECAS	24	75.0%	87.5%	100.0%	100.0%	91.7%	8.3%	95.8%	100.0%	12.5%	100.0%	100.0%
**CENTER SOUTH**												
CDMX	1053	77.1%	91.7%	97.6%	98.8%	66.6%	5.0%	98.9%	85.8%	13.8%	91.5%	95.5%
HIDALGO	57	78.9%	94.7%	98.2%	98.2%	87.7%	17.5%	96.5%	89.5%	3.5%	91.2%	98.2%
EAST MEXICO	28	100.0%	100.0%	100.0%	100.0%	85.7%	92.9%	89.3%	100.0%	14.3%	100.0%	100.0%
WEST MEXICO	36	91.7%	91.7%	94.4%	94.4%	77.8%	50.0%	91.7%	100.0%	0.0%	97.2%	100.0%
MORELOS	51	92.2%	92.2%	100.0%	100.0%	90.2%	17.6%	98.0%	96.1%	9.8%	96.1%	98.0%
PUEBLA	49	73.5%	98.0%	98.0%	98.0%	93.9%	2.0%	98.0%	95.9%	4.1%	98.0%	100.0%
TLAXCALA	47	78.7%	100.0%	100.0%	100.0%	91.5%	2.1%	97.9%	97.9%	19.1%	100.0%	100.0%
**SOUTH**												
CHIAPAS	42	33.3%	81.0%	100.0%	100.0%	83.3%	11.9%	100.0%	92.9%	16.7%	95.2%	100.0%
GUERRERO	42	81.0%	88.1%	100.0%	100.0%	85.7%	45.2%	100.0%	97.6%	0.0%	95.2%	97.6%
OAXACA	45	80.0%	97.8%	100.0%	100.0%	80.0%	31.1%	95.6%	100.0%	8.9%	97.8%	100.0%
TABASCO	39	87.2%	94.9%	100.0%	100.0%	100.0%	5.1%	100.0%	94.9%	17.9%	100.0%	100.0%
VERACRUZ	38	78.9%	94.7%	100.0%	100.0%	97.4%	2.6%	97.4%	94.7%	18.4%	94.7%	100.0%
**SOUTHEAST**												
CAMPECHE	47	72.3%	91.5%	100.0%	97.9%	85.1%	14.9%	95.7%	91.5%	0.0%	95.7%	100.0%
QUINTANA ROO	47	83.0%	95.7%	100.0%	100.0%	100.0%	6.4%	100.0%	93.6%	6.4%	97.9%	100.0%
YUCATAN	38	71.1%	97.4%	100.0%	100.0%	97.4%	18.4%	100.0%	94.7%	18.4%	97.4%	100.0%
average	—	0.7651818	0.94293939	0.99124242	0.98469697	0.8636667	0.2127879	0.97563636	0.93893939	0.08539394	0.96475758	0.9934242
SD	—	0.1451431	0.04321092	0.01528281	0.02929962	0.0922343	0.2033539	0.02585031	0.05358168	0.08377647	0.03303221	0.0116271

Supplementary Table S1. Seroprevalence percentages for each state in Mexico. Descriptive table showing the percentage of positive results from the ELISA assays against the 11 indicated AdV types (Figure 1). Results are shown for each state and are grouped by geographical region.

	Neutralization (%)										
	N	HAdV-A12	HAdV-B14	HAdV-C5	HAdV-C6	HAdV-D36	HAdV-E4	HAdV-F41	HAdV-D26	SAdV-21	SAdV-25	SAdV-31
**NORTHEAST**												
COAHUILA	51	15.7%	78.4%	72.5%	76.5%	15.7%	21.6%	49.0%	15.7%	0.0%	0.0%	0.0%
NUEVO LEON	50	52.0%	90.0%	94.0%	94.0%	52.0%	30.0%	68.0%	24.0%	0.0%	0.0%	0.0%
TAMAULIPAS	49	73.5%	91.8%	93.9%	87.8%	38.8%	49.0%	87.8%	6.1%	0.0%	0.0%	0.0%
**NORTHWEST**												
BAJA CALIFORNIA	45	26.7%	80.0%	44.4%	42.2%	0.0%	31.1%	77.8%	0.0%	0.0%	0.0%	0.0%
BAJA CALIFORNIA SUR	50	28.0%	82.0%	30.0%	28.0%	0.0%	34.0%	70.0%	0.0%	0.0%	0.0%	0.0%
CHIHUAHUA	41	7.3%	56.1%	61.0%	80.5%	2.4%	7.3%	51.2%	0.0%	0.0%	0.0%	0.0%
DURANGO	46	89.1%	10.9%	97.8%	97.8%	87.0%	95.7%	84.8%	91.3%	0.0%	0.0%	0.0%
SINALOA	54	1.9%	79.6%	100.0%	100.0%	18.5%	9.3%	64.8%	3.7%	0.0%	0.0%	0.0%
SONORA	46	10.9%	69.6%	95.7%	89.1%	58.7%	2.2%	67.4%	10.9%	0.0%	0.0%	0.0%
**WEST**												
COLIMA	34	47.1%	91.2%	100.0%	100.0%	94.1%	38.2%	91.2%	94.1%	0.0%	0.0%	0.0%
JALISCO	51	68.6%	98.0%	96.1%	96.1%	56.9%	78.4%	92.2%	27.5%	0.0%	0.0%	0.0%
MICHOACAN	37	94.6%	100.0%	100.0%	94.6%	45.9%	94.6%	94.6%	10.8%	0.0%	0.0%	0.0%
NAYARIT	45	57.8%	97.8%	97.8%	97.8%	62.2%	46.7%	93.3%	13.3%	0.0%	0.0%	0.0%
**CENTER NORTH**												
AGUASCALIENTES	52	46.2%	84.6%	44.2%	53.8%	9.6%	21.2%	84.6%	0.0%	0.0%	0.0%	0.0%
GUANAJUATO	57	66.7%	84.2%	96.5%	94.7%	84.2%	91.2%	70.2%	66.7%	0.0%	0.0%	0.0%
QUERETARO	46	26.1%	87.0%	80.4%	73.9%	19.6%	4.3%	52.2%	10.9%	0.0%	0.0%	0.0%
SAN LUIS POTOSI	51	19.6%	60.8%	96.1%	92.2%	37.3%	11.8%	70.6%	11.8%	0.0%	0.0%	0.0%
ZACATECAS	24	45.8%	79.2%	95.8%	87.5%	29.2%	33.3%	87.5%	4.2%	0.0%	0.0%	0.0%
**CENTER SOUTH**												
CDMX	1053	43.5%	81.6%	86.5%	86.4%	43.0%	44.2%	78.0%	23.6%	0.0%	11.6%	9.5%
HIDALGO	57	7.0%	93.0%	98.2%	98.2%	73.7%	28.1%	84.2%	82.5%	0.0%	0.0%	0.0%
EAST MEXICO	28	100.0%	100.0%	92.9%	92.9%	42.9%	92.9%	100.0%	3.6%	0.0%	0.0%	0.0%
WEST MEXICO	36	91.7%	100.0%	91.7%	91.7%	50.0%	94.4%	100.0%	8.3%	0.0%	0.0%	0.0%
MORELOS	51	88.2%	92.2%	98.0%	98.0%	54.9%	54.9%	82.4%	25.5%	0.0%	0.0%	0.0%
PUEBLA	49	6.1%	87.8%	89.8%	87.8%	8.2%	0.0%	65.3%	51.0%	0.0%	0.0%	0.0%
TLAXCALA	47	68.1%	100.0%	83.0%	80.9%	53.2%	14.9%	95.7%	6.4%	0.0%	0.0%	0.0%
**SOUTH**												
CHIAPAS	42	11.9%	31.0%	78.6%	90.5%	0.0%	28.6%	64.3%	0.0%	0.0%	0.0%	0.0%
GUERRERO	42	35.7%	88.1%	100.0%	100.0%	78.6%	45.2%	95.2%	73.8%	0.0%	0.0%	0.0%
OAXACA	45	6.7%	84.4%	93.3%	91.1%	13.3%	40.0%	77.8%	24.4%	0.0%	0.0%	0.0%
TABASCO	39	46.2%	87.2%	92.3%	89.7%	71.8%	53.8%	94.9%	5.1%	0.0%	0.0%	0.0%
VERACRUZ	38	71.1%	92.1%	97.4%	97.4%	71.1%	34.2%	94.7%	15.8%	0.0%	0.0%	0.0%
**SOUTHEAST**												
CAMPECHE	47	25.5%	83.0%	51.1%	61.7%	0.0%	36.2%	76.6%	0.0%	0.0%	0.0%	0.0%
QUINTANA ROO	47	21.3%	83.0%	97.9%	93.6%	38.3%	40.4%	83.0%	8.5%	0.0%	0.0%	0.0%
YUCATAN	38	57.9%	94.7%	94.7%	94.7%	63.2%	34.2%	78.9%	10.5%	0.0%	0.0%	0.0%
average	—	0.4419697	0.8240303	0.8610909	0.8609394	0.4164545	0.4066364	0.7964242	0.2212121	0	0.003515152	0.002878788
SD	—	0.2977770	0.1906608	0.1875412	0.1689839	0.2851659	0.2825943	0.1418620	0.2797644	0	0.020193008	0.016537377

Supplementary Table S2. Neutralization percentages for each state in Mexico. Descriptive table showing the percentages of positive results from the neutralization assays against the 11 indicated AdV types (Figure 2). Results are shown for each state and grouped by geographical region. The average and standard deviation are indicated for each virus type.

Percent Identity Matrix Hexon

Human-adenovirus-12/1-919	100.00	82.18	73.93	83.11	73.60	73.41	80.02	80.87	78.45	78.52	76.76	78.87	78.60
Human-adenovirus-41/1-925	82.18	100.00	75.41	84.99	75.22	74.76	80.37	82.56	81.11	80.75	78.34	79.78	80.64
Human-adenovirus-6/1-963	73.93	75.41	100.00	75.19	81.97	81.62	74.10	76.31	75.19	75.49	75.16	75.57	75.11
Rhesus_adenovirus_63/1-931	83.11	84.99	75.19	100.00	74.21	73.27	80.19	81.91	79.01	79.15	78.38	79.33	79.40
Simian-adenovirus-31/1-954	73.60	75.22	81.97	74.21	100.00	86.50	73.99	75.88	75.19	75.27	74.33	75.25	75.98
Human-adenovirus-5/1-952	73.41	74.76	81.62	73.27	86.50	100.00	73.27	75.36	74.54	74.97	73.79	74.51	75.16
Human-adenovirus-26/1-952	80.02	80.37	74.10	80.19	73.99	73.27	100.00	90.39	83.50	83.39	85.43	84.19	84.03
Human-adenovirus-36/1-944	80.87	82.56	76.31	81.91	75.88	75.36	90.39	100.00	81.60	83.67	85.39	83.66	84.04
Human-adenovirus-14/1-944	78.45	81.11	75.19	79.01	75.19	74.54	83.50	81.60	100.00	92.67	82.14	85.43	83.19
Simian-adenovirus-21/1-956	78.52	80.75	75.49	79.15	75.27	74.97	83.39	83.67	92.67	100.00	82.28	85.75	83.93
Chimpanzee-adenovirus-Y25/1-942	76.76	78.34	75.16	78.38	74.33	73.79	85.43	85.39	82.14	82.28	100.00	85.88	84.64
Simian-adenovirus-25/1-933	78.87	79.78	75.57	79.33	75.25	74.51	84.19	83.66	85.43	85.75	85.88	100.00	86.91
Human-adenovirus-4/1-936	78.60	80.64	75.11	79.40	75.98	75.16	84.03	84.04	83.19	83.93	84.64	86.91	100.00

Percent Identity Matrix Fiber

Human-adenovirus-12/1-587	100.00	11.49	16.07	15.83	14.31	16.92	14.65	13.22	13.61	14.99	13.19	9.19	13.38	10.80	14.51
Chimpanzee-adenovirus-Y25/1-443	11.49	100.00	40.92	39.71	25.40	28.87	13.02	14.02	16.38	14.13	16.79	17.77	13.48	11.63	11.86
Simian-adenovirus-25/1-425	16.07	40.92	100.00	91.06	18.44	39.53	11.57	15.48	15.45	16.61	17.91	11.63	12.79	10.67	12.37
Human-adenovirus-4/1-425	15.83	39.71	91.06	100.00	18.20	37.98	11.28	15.48	15.00	15.87	17.16	11.88	13.70	11.17	12.01
Human-adenovirus-5/1-581	14.31	25.40	18.44	18.20	100.00	27.13	13.45	12.75	14.33	12.10	16.53	24.82	12.85	11.25	11.72
Human-adenovirus-6/1-528	16.92	28.87	39.53	37.98	27.13	100.00	12.58	12.18	17.45	15.05	15.18	11.83	13.79	13.38	10.16
Rhesus-adenovirus-63-Fiber-protein-1/1-529	14.65	13.02	11.57	11.28	13.45	12.58	100.00	13.17	13.99	13.35	14.37	12.80	14.74	13.06	12.29
Rhesus-adenovirus-63-Fiber-protein-2/1-359	13.22	14.02	15.48	15.48	12.75	12.18	13.17	100.00	11.29	18.03	10.99	7.10	13.17	11.58	15.45
Human-adenovirus-14/1-323	13.61	16.38	15.45	15.00	14.33	17.45	13.99	11.29	100.00	18.89	17.03	11.18	21.50	13.12	9.01
Human-adenovirus-26/1-374	14.99	14.13	16.61	15.87	12.10	15.05	13.35	18.03	18.89	100.00	25.07	10.99	15.26	7.55	12.06
Human-adenovirus-36/1-371	13.19	16.79	17.91	17.16	16.53	15.18	14.37	10.99	17.03	25.07	100.00	10.23	11.84	6.25	15.95
Simian-adenovirus-31/1-596	9.19	17.77	11.63	11.88	24.82	11.83	12.80	7.10	11.18	10.99	10.23	100.00	9.21	9.78	10.30
Simian-adenovirus-21/1-322	13.38	13.48	12.79	13.70	12.85	13.79	14.74	13.17	21.50	15.26	11.84	9.21	100.00	9.38	12.42
Human-adenovirus-41-Fiber-protein-1/1-562	10.80	11.63	10.67	11.17	11.25	13.38	13.06	11.58	13.12	7.55	6.25	9.78	9.38	100.00	11.17
Human-adenovirus-41-Fiber-protein-2/1-387	14.51	11.86	12.37	12.01	11.72	10.16	12.29	15.45	9.01	12.06	15.95	10.30	12.42	11.17	100.00

Percent Identity Matrix Penton

Human-adenovirus-41/1-508	100.00	79.88	77.03	77.91	79.60	78.83	77.82	87.25	75.35	75.35	76.00	78.01	78.42
Human-adenovirus-12/1-497	79.88	100.00	77.30	77.98	79.27	79.51	78.30	78.73	78.57	78.37	78.74	77.75	77.75
Human-adenovirus-14/1-558	77.03	77.30	100.00	86.00	85.85	86.38	84.14	75.66	71.00	70.84	72.34	81.24	82.40
Simian-adenovirus-21/1-564	77.91	77.98	86.00	100.00	87.76	88.65	86.81	76.75	72.07	71.53	73.80	81.11	82.47
Simian-adenovirus-25/1-534	79.60	79.27	85.85	87.76	100.00	98.68	95.51	77.98	76.65	76.64	77.84	79.48	79.88
Chimpanzee-adenovirus-Y25/1-532	78.83	79.51	86.38	88.65	98.68	100.00	95.86	77.43	75.92	75.92	77.65	80.08	79.88
Human-adenovirus-4/1-535	77.82	78.30	84.14	86.81	95.51	95.86	100.00	76.24	75.53	75.53	77.07	78.69	78.49
Rhesus-adenovirus-63/1-505	87.25	78.73	75.66	76.75	77.98	77.43	76.24	100.00	75.50	75.50	75.90	76.81	77.23
Human-adenovirus-5/1-570	75.35	78.57	71.00	72.07	76.65	75.92	75.53	75.50	100.00	91.23	83.45	74.95	73.91
Human-adenovirus-6/1-574	75.35	78.37	70.84	71.53	76.64	75.92	75.53	75.50	91.23	100.00	82.33	74.36	74.51
Simian-adenovirus-31/1-589	76.00	78.74	72.34	73.80	77.84	77.65	77.07	75.90	83.45	82.33	100.00	75.29	74.46
Human-adenovirus-36/1-520	78.01	77.75	81.24	81.11	79.48	80.08	78.69	76.81	74.95	74.36	75.29	100.00	90.17
Human-adenovirus-26/1-519	78.42	77.75	82.40	82.47	79.88	79.88	78.49	77.23	73.91	74.51	74.46	90.17	100.00

Supplementary Table S3. Sequence identity matrices for the Hexon, Fiber and Penton proteins of 13 human and simian adenoviruses. Pairwise percentage identities were calculated from multiple sequence alignments (as described in Materials and Methods). High sequence homology was observed among the different sequences, with simian adenoviruses clustering with human groups B, C, and E, consistent with the phylogenetic trees shown in Figure 3.

Percent Identity Matrix Hexon-Epitope (ETTEERQNEDGENDEKA)

1: Rhesus_adenovirus_63/69-93	100.00	5.88	16.00	4.00	12.00	4.00	0.00	12.00	8.00	0.00	0.00	12.00	8.00
2: Human-adenovirus-14/69-85	5.88	100.00	35.29	11.76	17.65	17.65	0.00	11.76	17.65	0.00	11.76	5.88	5.88
3: Simian-adenovirus-21/69-94	16.00	35.29	100.00	16.00	16.00	20.00	0.00	8.00	0.00	7.69	7.69	4.00	3.85
4: Human-adenovirus-6/69-93	4.00	11.76	16.00	100.00	16.00	24.00	11.76	8.00	12.00	8.00	4.00	8.00	4.00
5: Simian-adenovirus-31/69-93	12.00	17.65	16.00	16.00	100.00	48.00	0.00	8.00	4.00	0.00	0.00	8.00	4.00
6: Human-adenovirus-5/69-93	4.00	17.65	20.00	24.00	48.00	100.00	5.88	8.00	4.00	8.00	4.00	8.00	4.00
7: Human-adenovirus-36/69-85	0.00	0.00	0.00	11.76	0.00	5.88	100.00	17.65	17.65	17.65	11.76	52.94	11.76
8: Human-adenovirus-12/69-93	12.00	11.76	8.00	8.00	8.00	8.00	17.65	100.00	36.00	4.00	8.00	12.00	4.00
9: Human-adenovirus-41/69-93	8.00	17.65	0.00	12.00	4.00	4.00	17.65	36.00	100.00	0.00	4.00	8.00	8.00
10: Chimpanzee-adenovirus-Y25/69-94	0.00	0.00	7.69	8.00	0.00	8.00	17.65	4.00	0.00	100.00	50.00	12.00	11.54
11: Simian-adenovirus-25/69-94	0.00	11.76	7.69	4.00	0.00	4.00	11.76	8.00	4.00	50.00	100.00	12.00	3.85
12: Human-adenovirus-26/69-93	12.00	5.88	4.00	8.00	8.00	8.00	52.94	12.00	8.00	12.00	12.00	100.00	24.00
13: Human-adenovirus-4/69-94	8.00	5.88	3.85	4.00	4.00	4.00	11.76	4.00	8.00	11.54	3.85	24.00	100.00

Percent Identity Matrix Hexon-Epitope (LEVPAEGDP)

1: Human-adenovirus-12/168-170	100.00	0.00	0.00	0.00	33.33	0.00	0.00	0.00	0.00	33.33	33.33	100.00	0.00
2: Human-adenovirus-6/171-174	0.00	100.00	33.33	33.33	0.00	0.00	0.00	0.00	0.00	0.00	0.00	0.00	0.00
3: Simian-adenovirus-31/171-174	0.00	33.33	100.00	100.00	0.00	0.00	0.00	33.33	33.33	0.00	0.00	-nan	0.00
4: Human-adenovirus-5/171-174	0.00	33.33	100.00	100.00	0.00	0.00	0.00	33.33	33.33	0.00	0.00	-nan	0.00
5: Chimpanzee-adenovirus-Y25/168-174	33.33	0.00	0.00	0.00	100.00	42.86	28.57	0.00	0.00	0.00	0.00	0.00	0.00
6: Simian-adenovirus-25/168-174	0.00	0.00	0.00	0.00	42.86	100.00	28.57	14.29	14.29	0.00	0.00	0.00	0.00
7: Human-adenovirus-4/168-174	0.00	0.00	0.00	0.00	28.57	28.57	100.00	14.29	14.29	0.00	0.00	0.00	0.00
8: Human-adenovirus-14/181-189	0.00	0.00	33.33	33.33	0.00	14.29	14.29	100.00	88.89	11.11	22.22	0.00	0.00
9: Simian-adenovirus-21/190-198	0.00	0.00	33.33	33.33	0.00	14.29	14.29	88.89	100.00	11.11	11.11	0.00	0.00
10: Human-adenovirus-36/175-183	33.33	0.00	0.00	0.00	0.00	0.00	0.00	11.11	11.11	100.00	44.44	50.00	50.00
11: Human-adenovirus-26/176-184	33.33	0.00	0.00	0.00	0.00	0.00	0.00	22.22	11.11	44.44	100.00	0.00	25.00
12: Human-adenovirus-41/168-169	100.00	0.00	-nan	-nan	0.00	0.00	0.00	0.00	0.00	50.00	0.00	100.00	50.00
13: Rhesus_adenovirus_63/171-174	0.00	0.00	0.00	0.00	0.00	0.00	0.00	0.00	0.00	50.00	25.00	50.00	100.00

Percent Identity Matrix Hexon-Epitope (GDQAWKDVNPNGI)

1: Human-adenovirus-14/429-441	100.00	58.33	25.00	16.67	14.29	14.29	7.69	15.38	7.69	0.00	23.08	15.38	7.69
2: Simian-adenovirus-21/442-453	58.33	100.00	25.00	16.67	14.29	14.29	8.33	16.67	8.33	0.00	16.67	25.00	16.67
3: Simian-adenovirus-25/427-430	25.00	25.00	100.00	25.00	50.00	50.00	25.00	0.00	25.00	25.00	25.00	0.00	0.00
4: Human-adenovirus-41/410-422	16.67	16.67	25.00	100.00	14.29	12.50	25.00	25.00	41.67	8.33	25.00	41.67	41.67
5: Human-adenovirus-4/427-433	14.29	14.29	50.00	14.29	100.00	71.43	28.57	28.57	42.86	28.57	28.57	28.57	0.00
6: Human-adenovirus-12/410-419	14.29	14.29	50.00	12.50	71.43	100.00	28.57	28.57	42.86	14.29	28.57	42.86	28.57
7: Human-adenovirus-36/429-441	7.69	8.33	25.00	25.00	28.57	28.57	100.00	23.08	30.77	30.77	23.08	30.77	15.38
8: Human-adenovirus-26/437-449	15.38	16.67	0.00	25.00	28.57	28.57	23.08	100.00	53.85	7.69	38.46	23.08	7.69
9: Chimpanzee-adenovirus-Y25/427-439	7.69	8.33	25.00	41.67	42.86	42.86	30.77	53.85	100.00	0.00	23.08	38.46	15.38
10: Human-adenovirus-5/437-449	0.00	0.00	25.00	8.33	28.57	14.29	30.77	7.69	0.00	100.00	38.46	15.38	0.00
11: Simian-adenovirus-31/439-451	23.08	16.67	25.00	25.00	28.57	28.57	23.08	38.46	23.08	38.46	100.00	38.46	7.69
12: Human-adenovirus-6/448-460	15.38	25.00	0.00	41.67	28.57	42.86	30.77	23.08	38.46	15.38	38.46	100.00	38.46
13: Rhesus_adenovirus_63/416-428	7.69	16.67	0.00	41.67	0.00	28.57	15.38	7.69	15.38	0.00	7.69	38.46	100.00

Percent Identity Matrix Hexon-Epitope (KETPSLGSGYDPYYTY)

1: Human-adenovirus-12/533-548	100.00	100.00	100.00	100.00	100.00	100.00	100.00	100.00	100.00	93.75	81.25	81.25	87.50
2: Rhesus_adenovirus_63/545-560	100.00	100.00	100.00	100.00	100.00	100.00	100.00	100.00	100.00	93.75	81.25	81.25	87.50
3: Human-adenovirus-26/566-581	100.00	100.00	100.00	100.00	100.00	100.00	100.00	100.00	100.00	93.75	81.25	81.25	87.50
4: Human-adenovirus-36/558-573	100.00	100.00	100.00	100.00	100.00	100.00	100.00	100.00	100.00	93.75	81.25	81.25	87.50
5: Human-adenovirus-14/558-573	100.00	100.00	100.00	100.00	100.00	100.00	100.00	100.00	100.00	93.75	81.25	81.25	87.50
6: Simian-adenovirus-21/570-585	100.00	100.00	100.00	100.00	100.00	100.00	100.00	100.00	100.00	93.75	81.25	81.25	87.50
7: Chimpanzee-adenovirus-Y25/556-571	100.00	100.00	100.00	100.00	100.00	100.00	100.00	100.00	100.00	93.75	81.25	81.25	87.50
8: Simian-adenovirus-25/547-562	100.00	100.00	100.00	100.00	100.00	100.00	100.00	100.00	100.00	93.75	81.25	81.25	87.50
9: Human-adenovirus-4/550-565	100.00	100.00	100.00	100.00	100.00	100.00	100.00	100.00	100.00	93.75	81.25	81.25	87.50
10: Human-adenovirus-41/539-554	93.75	93.75	93.75	93.75	93.75	93.75	93.75	93.75	93.75	100.00	87.50	87.50	93.75
11: Human-adenovirus-6/577-592	81.25	81.25	81.25	81.25	81.25	81.25	81.25	81.25	81.25	87.50	100.00	100.00	93.75
12: Human-adenovirus-5/566-581	81.25	81.25	81.25	81.25	81.25	81.25	81.25	81.25	81.25	87.50	100.00	100.00	93.75
13: Simian-adenovirus-31/568-583	87.50	87.50	87.50	87.50	87.50	87.50	87.50	87.50	87.50	93.75	93.75	93.75	100.00

Percent Identity Matrix Fiber-Epitope(PYDTETGPPTVPFL)

1: Human-adenovirus-26/16-28	100.00	100.00	23.08	38.46	38.46	27.27	30.77	30.77	41.67	25.00	33.33	30.77	30.77	30.77	22.22
2: Human-adenovirus-36/16-28	100.00	100.00	23.08	38.46	38.46	27.27	30.77	30.77	41.67	25.00	33.33	30.77	30.77	30.77	22.22
3: Human-adenovirus-12/23-35	23.08	23.08	100.00	38.46	38.46	36.36	30.77	38.46	33.33	41.67	25.00	38.46	38.46	38.46	44.44
4: Human-adenovirus-5/16-29	38.46	38.46	38.46	100.00	100.00	83.33	46.15	38.46	33.33	41.67	25.00	61.54	61.54	61.54	33.33
5: Human-adenovirus-6/16-29	38.46	38.46	38.46	100.00	100.00	83.33	46.15	38.46	33.33	41.67	25.00	61.54	61.54	61.54	33.33
6: Simian-adenovirus-31/34-45	27.27	27.27	36.36	83.33	83.33	100.00	54.55	36.36	40.00	60.00	30.00	63.64	63.64	63.64	28.57
7: Rhesus-adenovirus-63-Fiber-protein-1/49-61	30.77	30.77	30.77	46.15	46.15	54.55	100.00	38.46	41.67	33.33	41.67	69.23	69.23	69.23	44.44
8: Human-adenovirus-14/16-28	30.77	30.77	38.46	38.46	38.46	36.36	38.46	100.00	66.67	33.33	41.67	38.46	38.46	38.46	55.56
9: Simian-adenovirus-21/16-27	41.67	41.67	33.33	33.33	33.33	40.00	41.67	66.67	100.00	27.27	45.45	50.00	50.00	50.00	44.44
10: Human-adenovirus-41-Fiber-protein-2/16-27	25.00	25.00	41.67	41.67	41.67	60.00	33.33	33.33	27.27	100.00	25.00	33.33	33.33	33.33	22.22
11: Human-adenovirus-41-Fiber-protein-1/16-27	33.33	33.33	25.00	25.00	25.00	30.00	41.67	41.67	45.45	25.00	100.00	41.67	41.67	41.67	55.56
12: Chimpanzee-adenovirus-Y25/16-29	30.77	30.77	38.46	61.54	61.54	63.64	69.23	38.46	50.00	33.33	41.67	100.00	100.00	100.00	55.56
13: Simian-adenovirus-25/16-29	30.77	30.77	38.46	61.54	61.54	63.64	69.23	38.46	50.00	33.33	41.67	100.00	100.00	100.00	55.56
14: Human-adenovirus-4/16-29	30.77	30.77	38.46	61.54	61.54	63.64	69.23	38.46	50.00	33.33	41.67	100.00	100.00	100.00	55.56
15: Rhesus-adenovirus-63-Fiber-protein-2/16-24	22.22	22.22	44.44	33.33	33.33	28.57	44.44	55.56	44.44	22.22	55.56	55.56	55.56	55.56	100.00

Percent Identity Matrix Penton-Epitope (DFTPTEASTQTINFD)

1: Human-adenovirus-5/100-114	100.00	100.00	93.33	80.00	73.33	73.33	73.33	73.33	73.33	73.33	73.33	66.67	66.67
2: Human-adenovirus-6/100-114	100.00	100.00	93.33	80.00	73.33	73.33	73.33	73.33	73.33	73.33	73.33	66.67	66.67
3: Simian-adenovirus-31/93-107	93.33	93.33	100.00	80.00	73.33	73.33	73.33	73.33	73.33	73.33	73.33	66.67	66.67
4: Human-adenovirus-12/90-104	80.00	80.00	80.00	100.00	73.33	73.33	73.33	73.33	73.33	73.33	73.33	80.00	80.00
5: Human-adenovirus-36/87-101	73.33	73.33	73.33	73.33	100.00	100.00	93.33	93.33	93.33	93.33	93.33	93.33	86.67
6: Human-adenovirus-26/87-101	73.33	73.33	73.33	73.33	100.00	100.00	93.33	93.33	93.33	93.33	93.33	93.33	86.67
7: Human-adenovirus-14/101-115	73.33	73.33	73.33	73.33	93.33	93.33	100.00	100.00	100.00	100.00	100.00	86.67	93.33
8: Simian-adenovirus-21/101-115	73.33	73.33	73.33	73.33	93.33	93.33	100.00	100.00	100.00	100.00	100.00	86.67	93.33
9: Simian-adenovirus-25/95-109	73.33	73.33	73.33	73.33	93.33	93.33	100.00	100.00	100.00	100.00	100.00	86.67	93.33
10: Chimpanzee-adenovirus-Y25/96-110	73.33	73.33	73.33	73.33	93.33	93.33	100.00	100.00	100.00	100.00	100.00	86.67	93.33
11: Human-adenovirus-4/96-110	73.33	73.33	73.33	73.33	93.33	93.33	100.00	100.00	100.00	100.00	100.00	86.67	93.33
12: Human-adenovirus-41/98-112	66.67	66.67	66.67	80.00	93.33	93.33	86.67	86.67	86.67	86.67	86.67	100.00	93.33
13: Rhesus-adenovirus-63/96-110	66.67	66.67	66.67	80.00	86.67	86.67	93.33	93.33	93.33	93.33	93.33	93.33	100.00

Percent Identity Matrix Penton-Epitope (DFTPTEASTQTINFD)

1: Human-adenovirus-12/6-21	100.00	60.00	46.67	60.00	60.00	80.00	80.00	80.00	80.00	80.00	63.64	63.64	63.64
2: Rhesus-adenovirus-63/6-21	60.00	100.00	57.14	63.64	63.64	81.82	81.82	81.82	81.82	81.82	75.00	75.00	75.00
3: Human-adenovirus-41/6-21	46.67	57.14	100.00	55.56	55.56	88.89	88.89	88.89	88.89	88.89	80.00	80.00	80.00
4: Human-adenovirus-36/6-21	60.00	63.64	55.56	100.00	100.00	62.50	68.75	68.75	68.75	68.75	53.33	53.33	53.33
5: Human-adenovirus-26/6-21	60.00	63.64	55.56	100.00	100.00	62.50	68.75	68.75	68.75	68.75	53.33	53.33	53.33
6: Human-adenovirus-14/12-27	80.00	81.82	88.89	62.50	62.50	100.00	93.75	93.75	93.75	93.75	73.33	73.33	73.33
7: Simian-adenovirus-21/13-28	80.00	81.82	88.89	68.75	68.75	93.75	100.00	100.00	100.00	100.00	73.33	73.33	73.33
8: Simian-adenovirus-25/6-21	80.00	81.82	88.89	68.75	68.75	93.75	100.00	100.00	100.00	100.00	73.33	73.33	73.33
9: Chimpanzee-adenovirus-Y25/6-21	80.00	81.82	88.89	68.75	68.75	93.75	100.00	100.00	100.00	100.00	73.33	73.33	73.33
10: Human-adenovirus-4/6-21	80.00	81.82	88.89	68.75	68.75	93.75	100.00	100.00	100.00	100.00	73.33	73.33	73.33
11: Human-adenovirus-5/6-21	63.64	75.00	80.00	53.33	53.33	73.33	73.33	73.33	73.33	73.33	100.00	100.00	87.50
12: Human-adenovirus-6/6-21	63.64	75.00	80.00	53.33	53.33	73.33	73.33	73.33	73.33	73.33	100.00	100.00	87.50
13: Simian-adenovirus-31/6-21	63.64	75.00	80.00	53.33	53.33	73.33	73.33	73.33	73.33	73.33	87.50	87.50	100.00

Supplementary Table S4. Sequence identity matrices of reported B-cell epitopes in the hexon, penton, and fiber proteins of human adenoviruses.

Pairwise percentage identities were calculated from multiple sequence alignments restricted to previously reported B-cell epitopes (see Materials and Methods). For hexon, most epitopes mapped to hypervariable regions but still showed considerable conservation across several serotypes, consistent with antibody cross-reactivity. In contrast, penton epitopes displayed more limited conservation, with clustering patterns corresponding to adenovirus species, suggesting subgroup-restricted reactivity. For fiber, linear epitopes were less well conserved and poorly defined, consistent with the requirement of trimerization for recognition of conformational epitopes in the knob domain. Together, these matrices highlight the variable degree of epitope conservation across the three structural proteins, providing a molecular basis for both cross-reactive and type-specific antibody responses.
